# Differential regulation of the *α-globin* locus by Krüppel-like factor 3 in erythroid and non-erythroid cells

**DOI:** 10.1186/1471-2199-15-8

**Published:** 2014-05-16

**Authors:** Alister PW Funnell, Douglas Vernimmen, Wooi F Lim, Ka Sin Mak, Beeke Wienert, Gabriella E Martyn, Crisbel M Artuz, Jon Burdach, Kate GR Quinlan, Douglas R Higgs, Emma Whitelaw, Richard CM Pearson, Merlin Crossley

**Affiliations:** 1 School of Biotechnology and Biomolecular Sciences, University of New South Wales, Sydney, NSW 2052, Australia; 2 The Roslin Institute, University of Edinburgh, Easter Bush Campus, Midlothian EH25 9RG, UK; 3 MRC Molecular Haematology Unit, Weatherall Institute of Molecular Medicine, University of Oxford, John Radcliffe Hospital, Headington, Oxford OX3 9DS, UK; 4 La Trobe Institute for Molecular Science, La Trobe University, Melbourne, Victoria 3086, Australia

**Keywords:** KLF1, KLF3, Alpha globin, Globin gene regulation, Transcription factor

## Abstract

**Background:**

Krüppel-like Factor 3 (KLF3) is a broadly expressed zinc-finger transcriptional repressor with diverse biological roles. During erythropoiesis, KLF3 acts as a feedback repressor of a set of genes that are activated by Krüppel-like Factor 1 (KLF1). Noting that KLF1 binds *α-globin* gene regulatory sequences during erythroid maturation, we sought to determine whether KLF3 also interacts with the *α-globin* locus to regulate transcription.

**Results:**

We found that expression of a human transgenic *α-globin* reporter gene is markedly up-regulated in fetal and adult erythroid cells of *Klf3*^−/−^ mice. Inspection of the mouse and human *α-globin* promoters revealed a number of canonical KLF-binding sites, and indeed, KLF3 was shown to bind to these regions both *in vitro* and *in vivo*. Despite these observations, we did not detect an increase in endogenous murine *α-globin* expression in *Klf3*^*−/−*^ erythroid tissue. However, examination of murine embryonic fibroblasts lacking KLF3 revealed significant de-repression of *α-globin* gene expression. This suggests that KLF3 may contribute to the silencing of the *α-globin* locus in non-erythroid tissue. Moreover, ChIP-Seq analysis of murine fibroblasts demonstrated that across the locus, KLF3 does not occupy the promoter regions of the *α-globin* genes in these cells, but rather, binds to upstream, DNase hypersensitive regulatory regions.

**Conclusions:**

These findings reveal that the occupancy profile of KLF3 at the *α-globin* locus differs in erythroid and non-erythroid cells. In erythroid cells, KLF3 primarily binds to the promoters of the adult *α-globin* genes, but appears dispensable for normal transcriptional regulation. In non-erythroid cells, KLF3 distinctly binds to the *HS-12* and *HS-26* elements and plays a non-redundant, albeit modest, role in the silencing of *α-globin* expression.

## Background

Krüppel-like Factor 3 (KLF3/BKLF) belongs to the KLF family of transcription factors, of which there are 17 members with diverse biological roles in development and cellular differentiation [1,2]. KLFs are characterized by a highly homologous C-terminal DNA-binding domain, containing three C2H2 zinc fingers that direct binding to CACCC boxes and related GC-rich sequences in the control regions of target genes [3]. KLF3 is predominantly a transcriptional repressor which recruits a co-repressor complex containing C-terminal binding protein (CtBP) to facilitate silencing of its target genes [4]. KLF3 is broadly expressed and has been shown to have roles in several processes, including erythropoiesis [5,6], adipogenesis [7,8], muscle cell differentiation [9], and B cell development [10,11].

The *Klf3* gene is highly expressed in the red blood cell lineage due to the presence of an erythroid specific promoter, which is driven by a related KLF, Krüppel-like Factor 1 (KLF1) [12]. KLF1 is a master regulator of erythropoiesis, with functional roles in many facets of erythroid development, including red blood cell structure, heme biosynthesis and *globin* gene regulation [13,14]. Loss of KLF1 is embryonic lethal, with *Klf1*^−/−^ mice dying *in utero* from lethal β-thalassemia, due to a failure of activation of *β-globin* gene expression [15,16]. In addition to regulating the *β-globin* gene, KLF1 has been shown to bind the *α-globin* locus [17-19], as a component of a complex of factors recruited when looping of enhancer elements to the proximal promoter occurs and initiates high level gene expression [17,20]. Loss of KLF1 leads to reduced *α-globin* gene expression and chromosome looping [21], although these effects are notably less severe than the down-regulation of β-globin expression, possibly due to functional redundancy between other KLF family members and related SP (specificity protein) factors [17]. In regulating both the *α-globin* and *β-globin* loci, it is probable that KLF1 contributes to the maintenance of globin chain balance, which is critical for red blood cell function and viability.

Given that KLF3 is required for normal erythropoiesis and is known to repress a subset of KLF1-driven target genes [5], we investigated whether KLF3 can also bind and repress the *α-globin* gene. In support of this, we found that expression of a GFP reporter transgene, driven by the human *α-globin* promoter and regulatory elements [22] is significantly up-regulated in *Klf3*^−/−^ mice. Furthermore, inspection of the *α-globin* promoter revealed numerous KLF3 consensus recognition sites and we confirmed that KLF3 binds to this region both *in vitro* in electrophoretic mobility shift assays and *in vivo* by chromatin immunoprecipitation. However, despite demonstrating an *in vivo* interaction of KLF3 with the *α-globin* locus, we did not detect de-regulated endogenous *α-globin* expression in *Klf3*^−/−^ erythroid tissue. In contrast, examination of *α-globin* mRNA levels in *Klf3*^−/−^ murine embryonic fibroblasts revealed a significant increase in expression. In fibroblasts, KLF3 was found to bind not at *α-globin* promoter regions, but at the upstream *HS-12* and *HS-26* regulatory regions. Together, these results suggest that KLF3 may have a role in the silencing of the *α-globin* locus in non-erythroid tissue.

## Methods

### Mouse lines

The generation of GFP Line3 [22] and *Klf3*^−/−^[8] lines have been described previously. Mice were maintained on the FVBN/J background and animal work was carried out under the approval of the Animal Care and Ethics Committees of the University of Sydney (project numbers L02/1-2005/3/4048, L02/6-2006/3/4344 and L02/7-2009/3/5079) and the University of New South Wales (approval number 09/128A).

### Cell sorting and flow cytometry

Flow cytometry was performed using a FACSCalibur Flow Cytometer (BD Biosciences, San Jose, CA) and data were analyzed using CellQuest Pro (BD Biosciences) or FlowJo v7.6.5 software (TreeStar, Ashland, OR). TER119 antibody was supplied by BD Biosciences and titrated to optimal concentration. TER119^+^ cells were purified from embryonic day 14.5 fetal liver (*Klf3*^+/+^, *Klf3*^+/−^ and *Klf3*^−/−^ littermates) using Magnetic Activated Cell Sorting with Anti-TER119 MicroBeads (Miltenyi Biotec Australia Pty Ltd, Macquarie Park, NSW, Australia) by positive selection using MS columns as per the supplier’s instructions.

### Cell culture

Mouse and human primary erythroblasts, murine erythroleukemia (MEL) cells and interspecific MEL hybrids (containing a copy of human chromosome 16) were cultured and differentiated as previously described [17]. K562 cells were cultured at 37°C in RPMI medium and COS-7 cells were cultured in Dulbecco’s Modified Eagle Medium (DMEM), each supplemented with 10% (v/v) fetal calf serum (FCS) and 1% (v/v) penicillin, streptomycin and glutamine solution (PSG) (Gibco-BRL Life Technologies, Grand Island NY). Murine embryonic fibroblasts (MEFs) were prepared from littermate E12.5 embryos (*Klf3*^+/+^, *Klf3*^+/−^ and *Klf3*^−/−^). Briefly, heart, liver, intestinal, lung and brain tissue were removed and remaining embryonic tissue was homogenized in 3 mL trypsin/EDTA using an 18-gauge needle. MEFs were subsequently incubated for 2–3 minutes at 37°C and were then transferred to 100 mm plates containing 7 mL DMEM (10% FCS, 1% PSG). The cells were then left undisturbed for 48 h at 37°C and were passaged every 2–3 days. MEF cells (passage 2 or 3) were immortalized by transfecting with 5 μg pRSV-T [23] using the FuGENE6 transfection reagent protocol (Roche Diagnostics Australia Pty Ltd, Castle Hill, NSW, Australia). Immortalized *Klf3*^−/−^ MEFs that have been stably rescued with KLF3-V5, or pMSCVpuro empty vector (Clontech Laboratories, Mountain View, CA) as a negative control, have been described previously [24].

### RNA extraction and cDNA synthesis

RNA extraction was performed using TRI-Reagent, according to the manufacturer’s guidelines (Sigma, St. Louis, MO). RNA samples were further purified using RNeasy columns (Qiagen, Victoria, Australia) and by treating with DNase I (Ambion, Austin, TX). Subsequently, cDNA was prepared using Superscript VILO cDNA synthesis kit (Invitrogen, Carlsbad, CA), according to the manufacturer’s instructions.

### Primers and real-time RT-PCR

Primer sequences for real-time RT-PCR were: mouse *α-globin*, 5′-GTCACGGCAAGAAGGTCGC-3′ and 5′-GGGGTGAAATCGGCAGGGT-3′; mouse *β-actin*, 5′-GCTTCTTTGCAGCTCCTTCGT-3′ and 5′- CCAGCGCAGCGATATCG-3′; mouse *18S*, 5′-CACGGCCGGTACAGTGAAAC-3′ and 5′-AGAGGAGCGAGCGACCAA-3′; mouse *Gapdh*, 5′-GTCTCCTGCGACTTCAGC-3′ and 5′-TCATTGTCATACCAGGAAATGAGC-3′; and as described previously for *Klf3*, *Klf8* and *Fam132a*[7,12,25]. Quantitative real-time PCR was performed using *Power* SYBR Green PCR Master Mix and the 7500 Fast Real-Time PCR System (Applied Biosystems, Foster City, CA), as described previously [26]. Data were analyzed using 7500 Software v2.0.4 (Applied Biosystems).

### Electrophoretic mobility shift assays (EMSAs)

EMSAs were carried out as described previously [27]. COS-7 cells in 100 mm plates were transfected with 5 μg vector (pMT3-empty or pMT3-Klf3 [28]) using FuGENE6 (Roche Diagnostics Australia Pty Ltd) as per the manufacturer’s protocol. Nuclear extracts from COS-7, uninduced K562, uninduced MEL and MEF cell lines were harvested as previously described [28]. Oligonucleotides used in the synthesis of radiolabelled probes were: human *α-globin* promoter, 5′-CGCAGGCCCCGCCCGGGACTC-3′ and 5′-GAGTCCCGGGCGGGGCCTGCG-3′; mouse *α-globin* promoter, 5′-TGGAGGACACGCCCTTGGAGG-3′ and 5′-CCTCCAAGGGCGTGTCCTCCA-3′; mouse *HS-26* probe 1, 5′-AGGTGTACACACCCAGGCCAA-3′ and 5′-TTGGCCTGGGTGTGTACACCT-3′, and; *HS-26* probe 2, 5′-AGGCCAAGGGTGGAGCAGACCA-3′ and 5′-TGGTCTGCTCCACCCTTGGCCT-3′. Supershift recognition of KLF3 was achieved using specific antiserum that has been described previously [27]. Probe sequences were identified using CLC Main Workbench software version 6.6.2 (CLC Bio, Cambridge, MA).

### Chromatin immunoprecipitation (ChIP)

ChIP assays were carried out as previously described [17,29], using the previously described anti-KLF3 antibody [27]. KLF3 ChIP-Seq analysis has previously been described [24] and enrichment tracks were visualized using Integrative Genomics Viewer [30].

### Western blotting

Western blots of nuclear extracts from MEF, MEL and COS-7 cells were performed as previously described [31] using KLF3 anti-serum [27]. Full-Range Rainbow Molecular Weight Marker was supplied by GE Healthcare (Piscataway, NJ).

## Results

### KLF3 regulates expression of a human transgenic *α-globin* promoter *in vivo*

To begin our investigation into potential regulation of the *α-globin* gene by KLF3, we made use of an existing well-characterized transgenic mouse model, termed Line3, in which a GFP reporter gene is expressed under the control of the human *α-globin* proximal promoter and *HS-40* enhancer region [22]. The red blood cells of Line3 mice express GFP and it is possible to accurately measure the level of expression by flow cytometry in either adult peripheral blood or erythroid cells purified from tissues, such as the fetal liver. To determine whether KLF3 has a role in regulating expression of the reporter gene, we introduced the homozygous transgene into *Klf3*^−/−^ mice [8] by breeding and compared GFP expression in *Klf3*^+/+^, *Klf3*^+/−^ and *Klf3*^−/−^ erythrocytes.

**Figure 1:**
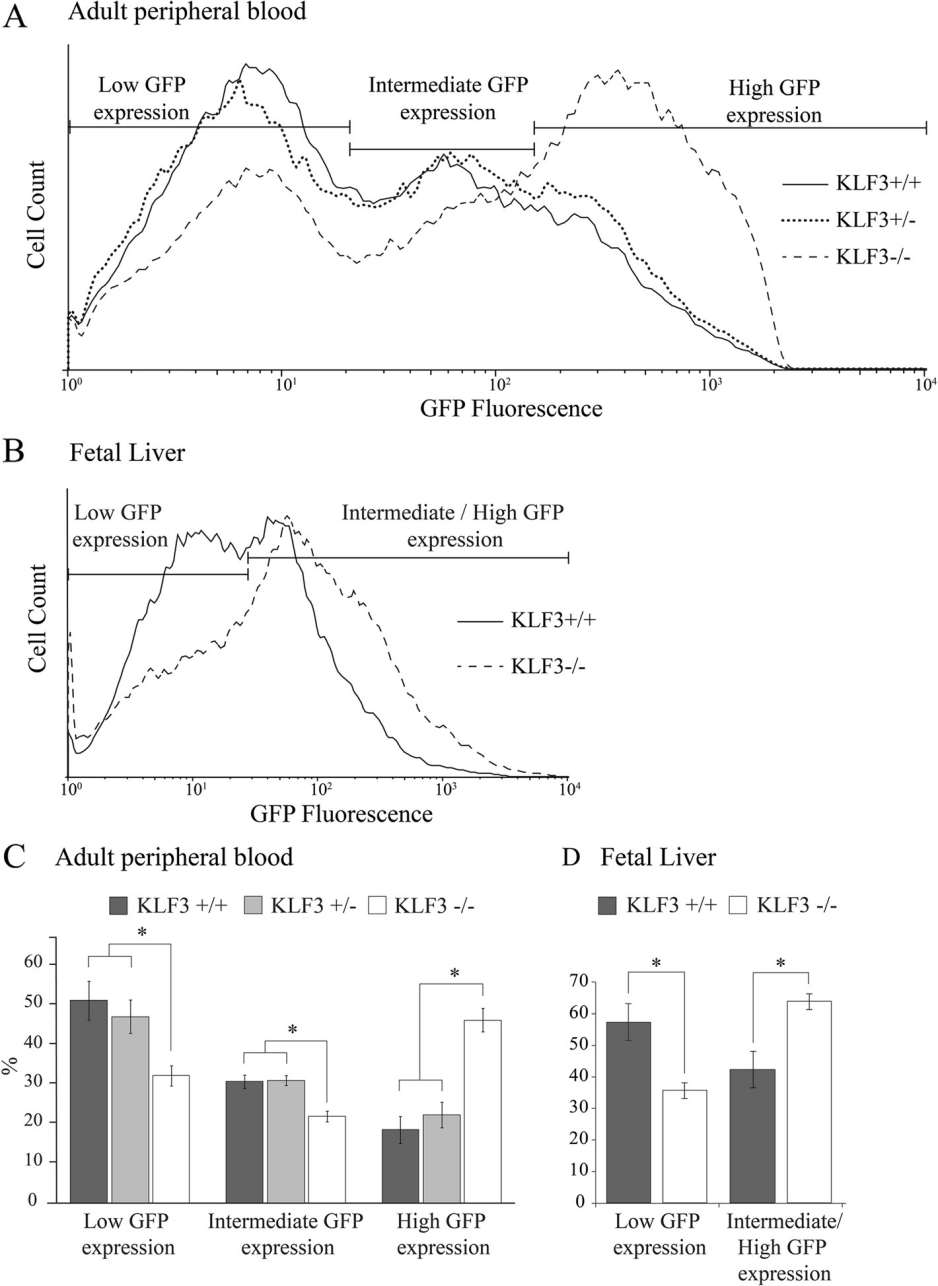
**Loss of KLF3 results in up-regulation of the human *α-globin *gene in a transgenic mouse model.** Line3 mice, containing a GFP transgene under the control of the human *α-globin* proximal promoter and *HS-40* enhancer [22], were crossed with *Klf3*^+/−^ mice to generate Line3::*Klf3*^+/+^, Line3::*Klf3*^+/−^ and Line3::*Klf3*^−/−^ mice, all homozygous for the transgene. Erythroid GFP fluorescence was then measured by flow cytometry. Shown are representative fluorescence profiles of **(A)** peripheral blood from mice at 3 weeks of age and **(B)** TER119^+^ sorted erythrocytes from embryonic day E14.5 fetal liver. The populations were gated to identify cells expressing low, intermediate and high levels of GFP. Statistical analysis of these gated populations is shown for **(C)** erythrocytes from mice at 3 weeks of age and (D) TER119^+^ fetal liver cells. For erythrocytes analyzed at 3 weeks of age, n = 32 for Line3::*Klf3*^+/+^, n = 48 for Line3::*Klf3*^+/−^ and n = 8 for Line3::*Klf3*^−/−^. For the analysis of fetal erythrocytes, n = 3 for Line3::*Klf3*^+/+^and n = 4 for Line3::*Klf3*^−/−^. Error bars represent standard deviation and * represents *P* < 0.05 (two tailed t-test).

As previously reported [22], we found that GFP is expressed in Line3::*Klf3*^+/+^erythrocytes with a broad, but consistent and reproducible profile. These cells can be classified as expressing low, intermediate or high levels of GFP (Figure 1). Loss of a single allele of *Klf3* had no effect on transgene expression, as we did not find any notable difference between the GFP profiles of Line3::*Klf3*^+/+^and Line3::*Klf3*^+/−^ mice (Figure 1A and 1C). However, analysis of red blood cells from homozygous Line3::*Klf3*^−/−^ animals revealed a significant increase in GFP expression (Figure 1A). On average, we found that 46% of *Klf3*^−/−^ cells express high levels of GFP, compared to 18% in *Klf3*^+/+^animals (Figure 1C). We also examined newly formed erythrocytes in the erythroid fetal liver. We purified TER119+ cells from the fetal livers of Line3::*Klf3*^+/+^and Line3::*Klf3*^−/−^ mice and again observed a significant increase in transgenic promoter activity in the absence of KLF3 (Figure 1B and 1D). Together, these data suggest that KLF3 directly or indirectly represses the human transgenic *α-globin* promoter in this mouse model.

### KLF3 binds the human and mouse *α-globin* promoters *in vitro* and *in vivo*

**Figure 2:**
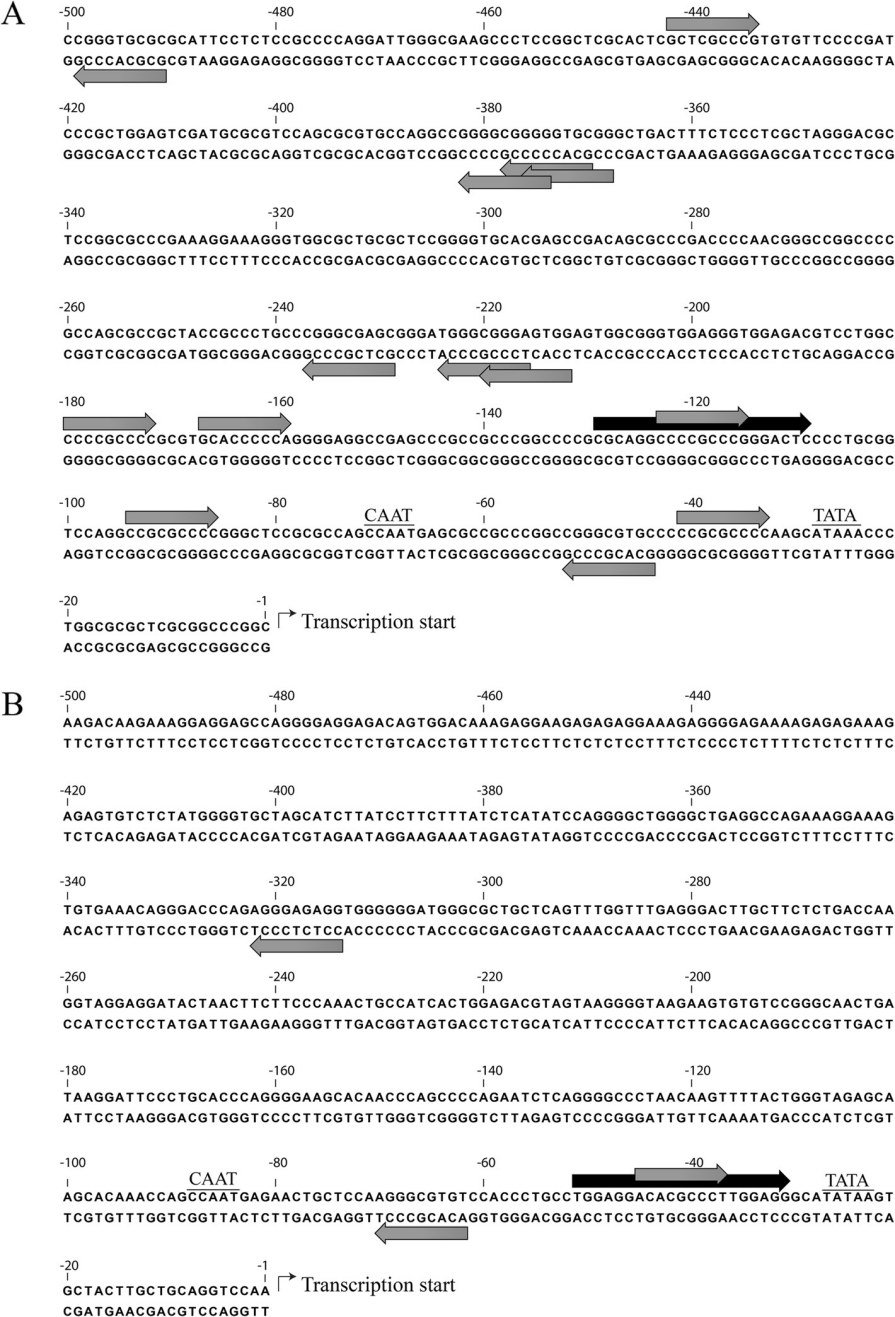
**The *α-globin *promoter contains many consensus KLF3 binding sites.** The human *HBA2***(A)** and mouse *Hba-a2***(B)***α-globin* proximal promoter sequences, immediately 5′ to the transcriptional start site, were inspected for consensus binding sites, conforming to the sequence 5′-NCN CNC CCN-3′. The position and direction of binding sites are indicated by grey arrows. The sequences used in the design of probes for electrophoretic mobility shift assays are shown by black arrows. Also indicated are CAAT and TATA boxes. Sequences are numbered with respect to the transcription start site at +1.

Having determined that KLF3 influences the expression of a transgene driven by *α-globin* gene regulatory sequences *in vivo*, we next investigated whether KLF3 interacts directly with the *α-globin* promoter. We inspected the human and mouse *α-globin* proximal promoters to identify potential high affinity KLF3 binding sites, which match the KLF consensus sequence, 5′-NCN CNC CCN-3′ [32]. This analysis revealed the presence of several sites, with the human promoter in particular containing 14 potential interaction motifs (Figure 2A and 2B). We then used our sequence analysis to design probes for electrophoretic mobility shift assays (EMSA) to investigate binding of KLF3 to the *α-globin* promoter *in vitro*. To assess binding to the human promoter, we based our probe on the most frequently seen consensus sequence, 5′-NCC CGC CCN-3′, which occurs four times (Figure 2A). In the case of the mouse promoter, where there are noticeably fewer potential KLF3 binding sites (Figure 2B), we used the sequence 5′-NCA CGC CCN-3′, which is found twice, to inform our probe design.

**Figure 3:**
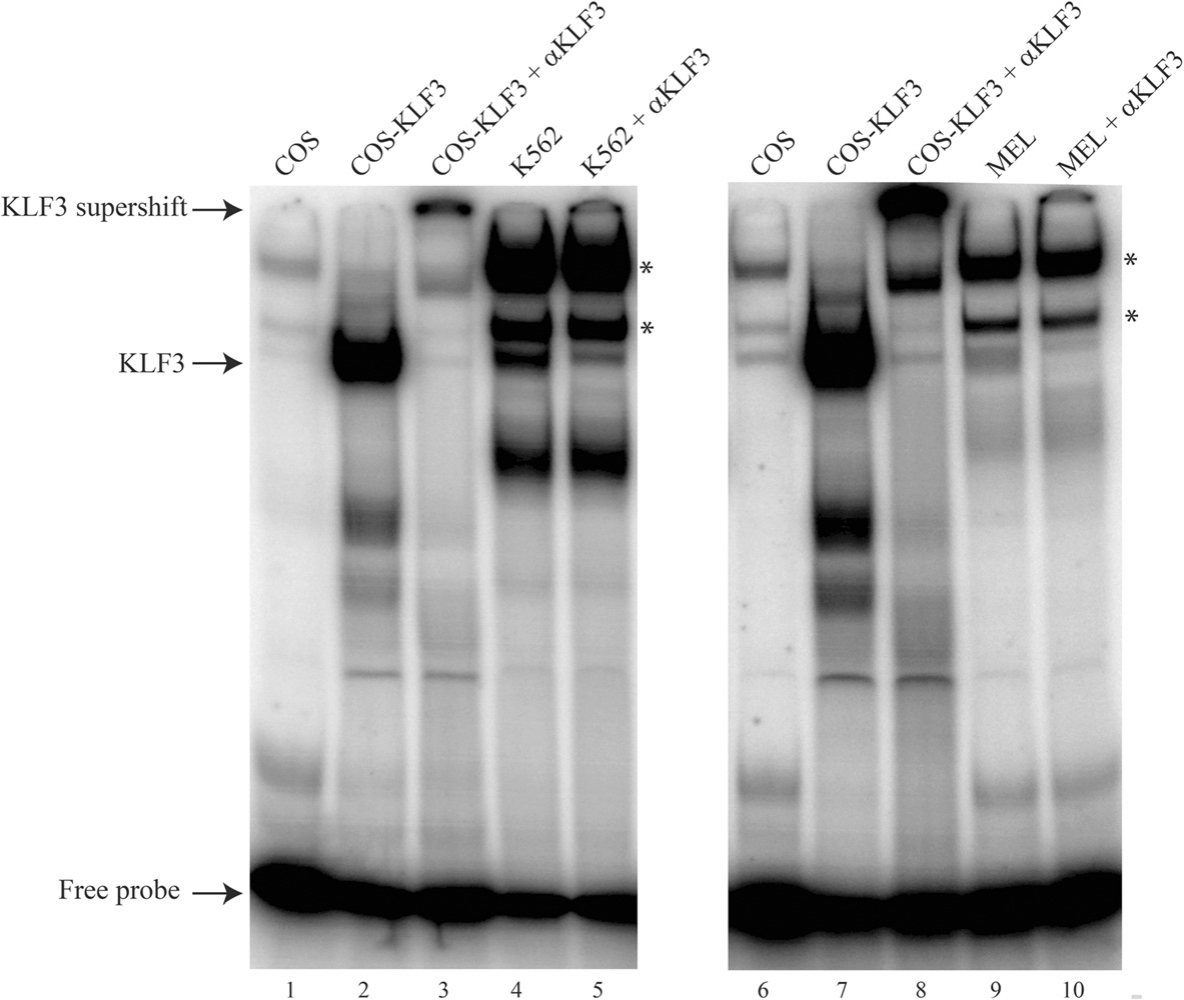
**KLF3 binds the *α-globin *promoter *in vitro*.** The binding of KLF3 to the *α-globin* promoter was assessed by EMSA, using radiolabeled probes designed from analysis of the human and mouse *α-globin* proximal promoter sequences (Figure 2). KLF3 was either expressed in COS-7 cells (lanes 2, 3, 7 and 8) or endogenous KLF3 was harvested in nuclear extracts from K562 (lanes 4 and 5) and MEL (lanes 9 and 10) erythroid cell lines. Nuclear extracts from mock transfected COS-7 cells have been included as a negative control (lanes 1 and 6). Binding to the human promoter sequence is shown in the left hand panel whilst binding to the mouse sequence is shown on the right. αKLF3 indicates an anti-KLF3 antibody used to validate KLF3 specific binding by supershift. Additional bands in lanes 4, 5, 9 and 10 (denoted by asterisks) most likely represent SP1 and SP3 as in [12].

We began our investigation into *in vitro* binding by expressing KLF3 in COS-7 cells and assessing the ability of nuclear extracts purified from these cells to interact with the human and mouse *α-globin* promoter sequences by EMSA. We found that the nuclear extracts bound both human and mouse probes with high affinity and confirmed that this interaction was specific to KLF3 by supershift with anti-KLF3 antibody (Figure 3, lanes 2–3 and 7–8). Minimal background binding was observed for nuclear extracts from mock transfected COS-7 cells (Figure 3, lanes 1 and 6). We next determined whether endogenous KLF3 present in erythroid cell lines also binds to the human and mouse *α-globin* promoter probes by preparing nuclear extracts from human K562 and murine erythroleukemia (MEL) cells. We tested binding of the K562 nuclear extracts to the human *α-globin* probe and the MEL nuclear extracts to the mouse probe. Again, we found that proteins in both extracts bound to the promoter sequences and confirmed the identity of a KLF3 complex by supershift with an anti-KLF3 antibody (Figure 3, lanes 4–5 and 9–10).

Having established that KLF3 can bind to both the human and mouse *α-globin* proximal promoters *in vitro*, we carried out chromatin immunoprecipitation (ChIP) assays on a number of erythroid cell types to determine whether KLF3 binds to the *α-globin* locus *in vivo*. Our approach was to conduct a primer walk across the locus, in which we used TaqMan real time RT-PCR probes to assess binding at the upstream HS (DNase hypersensitive) enhancers, the proximal promoter, the coding sequence, and at a number of control sites, including the *α-globin* intergenic region, and the *β-actin* and *β-globin* genes.

**Figure 4:**
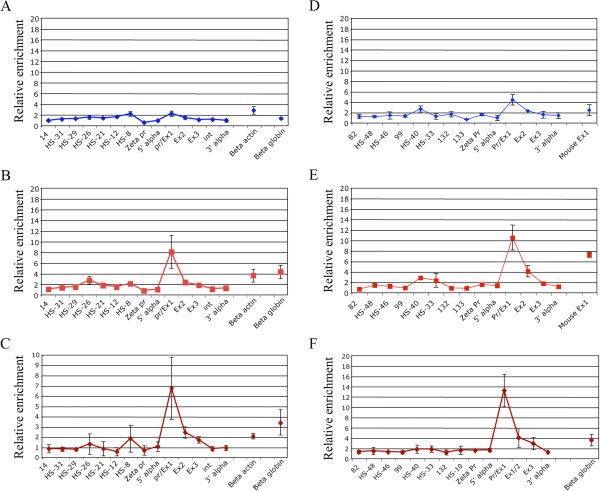
**KLF3 binds the human and mouse *α-globin *promoters *in vivo *in chromatin immunoprecipitation assays.** An anti-KLF3 antibody was used to immunoprecipitate chromatin from the following cell types: **(A)** uninduced MEL cells, **(B)** induced MEL cells, **(C)** mouse primary erythroblasts, **(D)** uninduced interspecific MEL hybrids containing a normal copy of human chromosome 16, **(E)** induced interspecific MEL hybrids, and **(F)** human primary erythroblasts. The y-axis represents enrichment over input DNA, normalized to a control sequence in the *Gapdh* gene (mouse) or *18S* (human). The x-axis represents the positions of the TaqMan probes used. The coding sequence is represented by the three exons (Promoter/Ex1, Ex2, and Ex3) of the *α-globin* genes. HS- primer sets refer to upstream DNase-hypersensitive regions. Zeta pr refers to the mouse and human embryonic *α-globin* promoters (*Hba-x* and *HBZ*). Inter, refers to the intergenic region (between mouse *Hba-a1* and *Hba-a2*). 5' and 3' are negative controls flanking the *α-globin* gene. β-actin and β-globin denote control sequences at the *β-actin* gene and *β-globin* promoter respectively. Error bars correspond to ±1 standard deviation from at least two independent ChIPs.

First, we investigated KLF3 binding to the *α-globin* locus in uninduced MEL cells and found only background binding at each of the sites we examined (Figure 4A). However, when we chemically induced erythroid maturation in these cells, we observed a marked enrichment of KLF3 at the *α-globin* proximal promoter (Figure 4B), consistent with what we have previously reported [6]. An examination of mouse primary erythroblasts confirmed that KLF3 also binds this site *in vivo* (Figure 4C). We then made use of an interspecies hybrid MEL cell line into which human chromosome 16, containing the *α-globin* locus, has been introduced [17,33]. Again, we saw only background binding of KLF3 across the locus in uninduced cells but observed noticeable enrichment at the human *α-globin* proximal promoter following erythroid maturation (Figure 4D and E). Finally, we assessed binding in human primary erythroblasts and once again found high enrichment at the *α-globin* proximal promoter (Figure 4F).

### KLF3 represses *α-globin* expression in non-erythroid tissue

**Figure 5:**
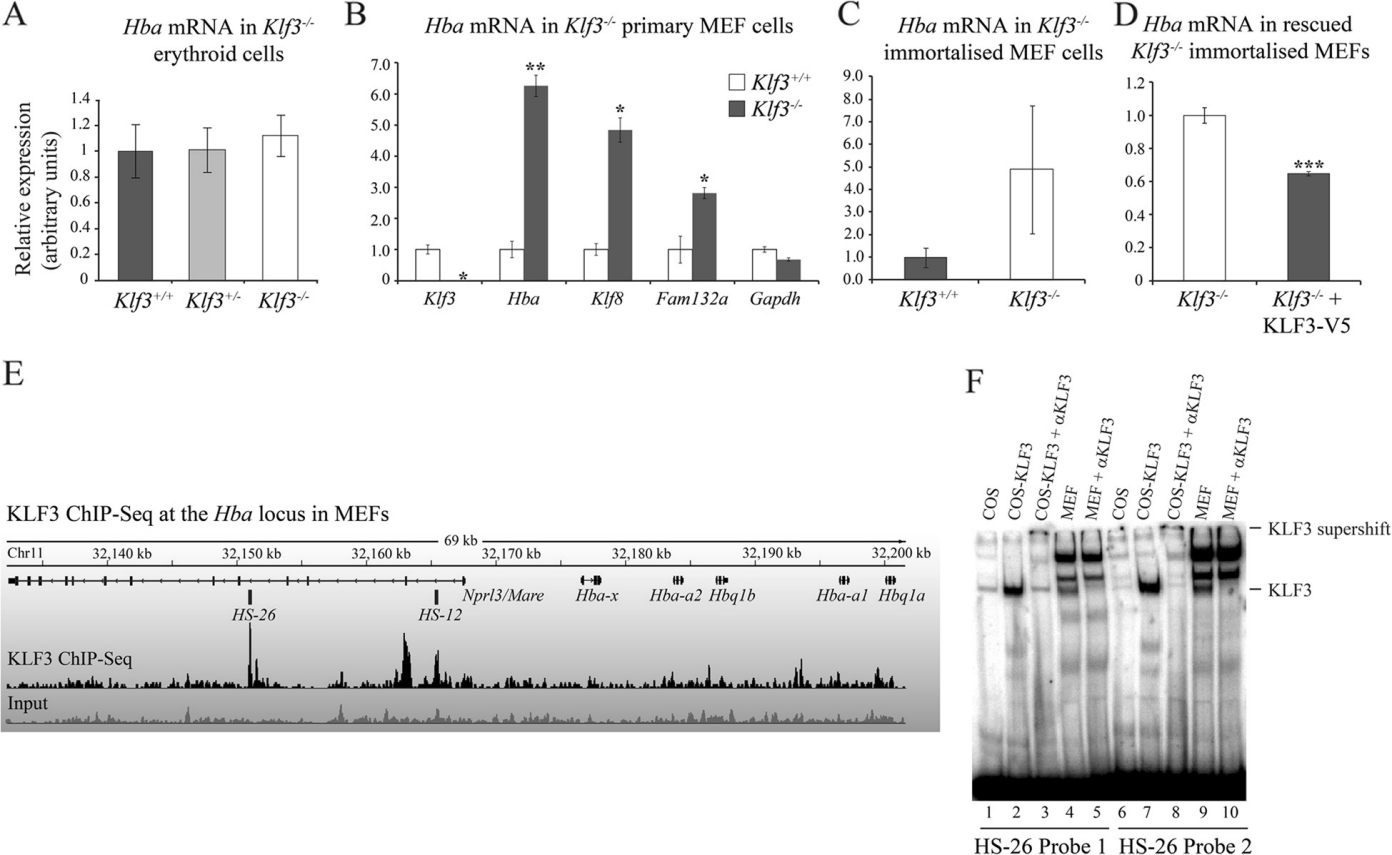
***α-globin *gene expression is de-repressed in murine embryonic fibroblasts lacking KLF3.***α-globin* mRNA expression levels were determined by real time qRT-PCR analysis of **(A)** TER119^+^ erythrocytes purified from embryonic day E14.5 fetal liver (*Klf3*^+/+^n = 3, *Klf3*^+/−^ n = 5, *Klf3*^−/−^ n = 6), **(B)** primary MEFs (*Klf3*^+/+^n = 2, *Klf3*^−/−^ n = 2 or 3), **(C)** immortalized MEFs (*Klf3*^+/+^n = 2, *Klf3*^−/−^ n = 2), and **(D)** immortalized *Klf3*^−/−^ MEFs rescued with KLF3-V5 or empty vector (n = 2 for each). **(A-D)** In each case, relative expression of *α-globin* mRNA was normalized to *18S* rRNA levels, and the expression levels of *Klf3*^+/+^**(A-C)** or *Klf3*^−/−^**(D)** were set to 1.0. In **(B)**, mRNA levels of *Klf3* and two known KLF3-repressed targets, *Klf8* and *Fam132a*[7,25], have also been analyzed together with a negative control, *Gapdh*. In **(A-D)**, error bars shown represent standard error of the mean, *P < 0.05 (one-tailed t-test relative to *Klf3*^+/+^), **P < 0.002 (two-tailed t-test relative to *Klf3*^+/+^), ***P < 0.05 (two-tailed t-test relative to *Klf3*^−/−^). **(E)** KLF3 ChIP-Seq track across the murine *α-globin* locus in MEFs from [24]. The positions of the *HS-12* and *HS-26* regulatory regions are indicated. **(F)** EMSA showing the binding of KLF3 to two sites within the *HS-26* region. Nuclear extracts were obtained from COS-7 cells that were mock-transfected (lanes 1 and 6) or transfected with pMT3-Klf3 (lanes 2, 3, 7 and 8). Nuclear extracts from MEFs are shown in lanes 4, 5, 9 and 10. Identification of KLF3:DNA complexes was achieved by addition of an antibody specific for KLF3 (αKLF3, lanes 3, 5, 8 and 10).

Having confirmed that KLF3 can bind to the *α-globin* promoter *in vitro* and *in vivo*, we next asked whether loss of KLF3 results in de-regulation of endogenous *α-globin* gene expression. We first compared *α-globin* mRNA levels in red blood cells purified from the erythroid fetal liver of *Klf3*^+/+^, *Klf3*^+/−^ and *Klf3*^−/−^ embryos (E14.5) by real time qRT-PCR. Despite our observation that KLF3 binds the *α-globin* gene promoter *in vivo*, we did not detect any up-regulation of *α-globin* expression in *Klf3*^−/−^ erythroid cells (Figure 5A). In addition, we have previously analyzed the expression of multiple *globin* genes at an earlier stage of development (E13.5) and similarly observed no change in adult *α-globin* transcripts in the absence of KLF3 [6].

It is possible that in erythroid cells, loss of KLF3 has little effect because *α-globin* is expressed at maximal levels. We therefore turned our attention to non-erythroid cells, namely murine embryonic fibroblasts (MEFs), which express only low levels of *α-globin* transcripts. In both primary and immortalized MEFs lacking KLF3, we observed a modest de-repression of *α-globin* gene expression (by 6.3-fold and 4.9-fold respectively compared to *Klf3*^+/+^cells) (Figure 5B and 5C). Furthermore, stable rescue of *Klf3*^−/−^ MEFs with V5-tagged KLF3 resulted in a significant diminution of *α-globin* mRNA expression (Figure 5D).

To explore KLF3’s potential mode of regulation at the *α-globin* locus in non-erythroid cells, we analyzed recently generated KLF3 ChIP-Seq data from MEF cells [24]. We found that in these cells, KLF3 was not bound to the adult *α-globin* promoters (*Hba-a1* and *Hba-a2*), but showed significant occupancy at the upstream *HS-12* and *HS-26* regulatory regions (Figure 5E). This contrasted with our observation from a series of erythroid cells (Figure 4), in which KLF3 was primarily found at the *α-globin* promoter. Analysis of the *HS-26* region revealed two sites resembling the KLF binding consensus via which KLF3 might be recruited. Indeed, EMSA experiments confirmed that both of these sites are recognized by both KLF3 expressed in COS-7 cells and endogenous KLF3 in MEFs (Figure 5F). Taken together, these findings suggest that in non-erythroid cells, KLF3 binds the *HS-12* and *HS-26* regulatory regions and may be involved in repressing and thereby maintain physiologically low levels of *α-globin* expression in these cells.

**Figure 6:**
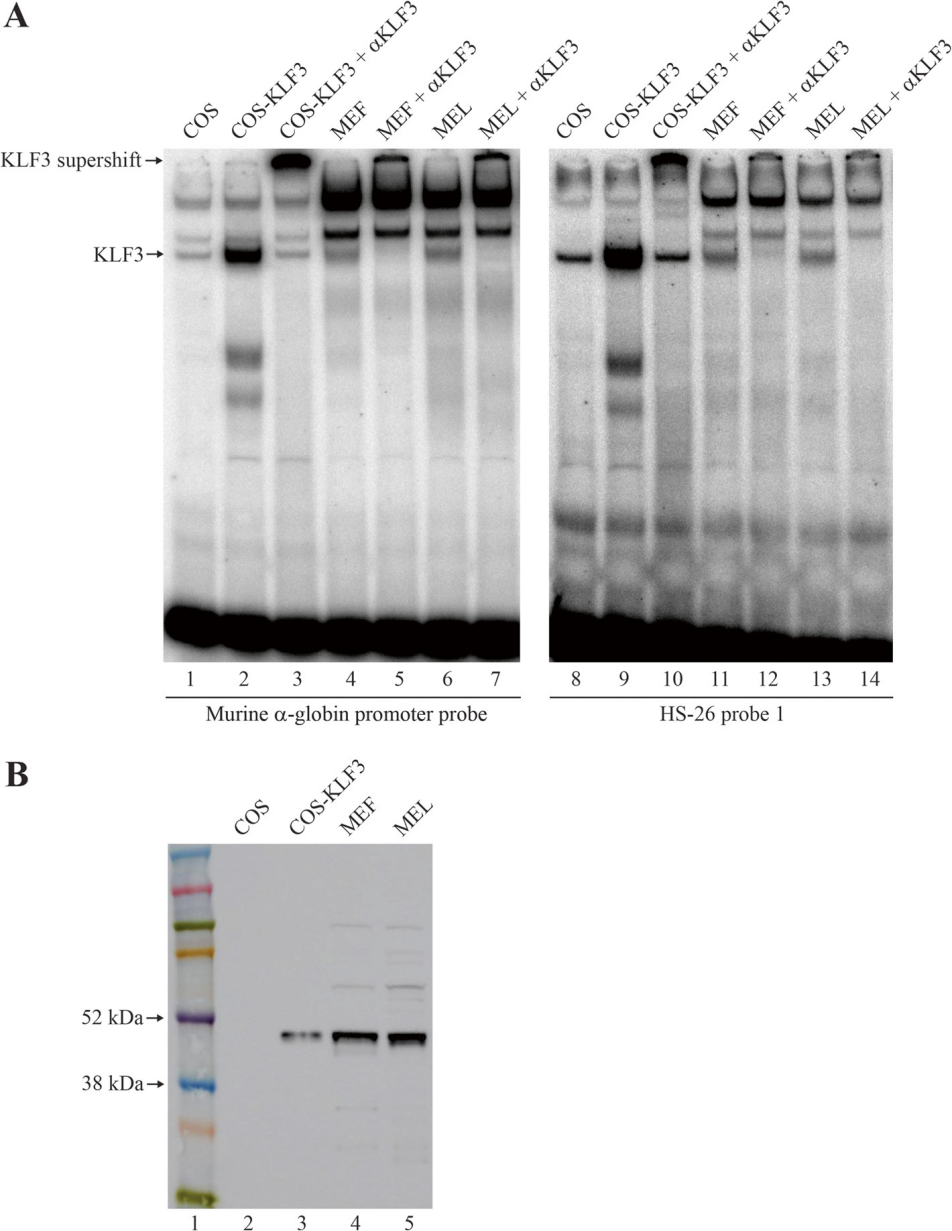
**KLF3 from erythroid and non-erythroid cells display similar DNA-binding abilities *in vitro*. (A)** EMSAs were employed to assess the binding of KLF3 to the murine *α-globin* promoter (lanes 1–7) and a site in the *HS-26* element from Figure 5 (lanes 8–14). Nuclear extracts were harvested from non-erythroid MEF (lanes 4, 5, 11 and 12) or erythroid MEL cells (lanes 6, 7, 13 and 14). Nuclear extracts from mock transfected COS-7 cells (lanes 1 and 8) or cells expressing KLF3 (lanes 2, 3, 9 and 10) were included as negative and positive controls respectively. The identity of KLF3 was confirmed by specific antibody supershifts (lanes 3, 5, 7, 10, 12 and 14). **(B)** Western blot demonstrating the relative amounts of KLF3 in MEF (lane 4) and MEL (lane 5) nuclear extracts used in the EMSAs in (A). As negative and positive controls, COS and COS-KLF3 nuclear extracts have been included (lanes 2 and 3) at 20-fold lower relative amounts than in (A) to facilitate visualization. A size ladder is shown in lane 1.

Lastly, we also analyzed the DNA-binding capacity of KLF3 extracted from erythroid (MEL) and non-erythroid (MEF) cells (Figure 6A and B). Equivalent levels of KLF3 from these two cellular sources exhibited comparable DNA-binding activity at sites in both the murine *α-globin* promoter and the *HS-26* regulatory element. This suggests that the differing *in vivo* occupancy of KLF3 across the *α-globin* locus in erythroid and non-erythroid cells (compare Figures 4B and 4C with Figure 5E) is not due to intrinsic differences in KLF3’s ability to bind DNA.

## Discussion

Our data show that KLF3 binds the adult mouse *α-globin* promoter in erythroid tissue *in vivo*. However, KLF3 does not appear to functionally repress the endogenous promoter in red blood cells. Similarly, we have previously observed KLF3 occupancy at the adult *β-globin* (*Hbb-b1*) promoter in erythroid cells and no associated perturbation of *Hbb-b1* transcription upon ablation of KLF3 [6]. It is notable that KLF3 binding is highest at the late stages of erythroid maturation (compare Figure 4A with 4B, and 4D with 4E) when the adult *globin* genes are expressed at very high levels and their promoters are presumably highly accessible. This is also when KLF3 levels peak [5] and it is possible that KLF3 gains access to these regions but is not sufficiently potent to limit KLF1 driven activation of the genes. This observation highlights the view that transcription factor binding sites discovered by ChIP may not always have functional relevance in the context in which they are identified, but may instead reflect the dynamic nature of transcription factor binding at permissive loci. Indeed, a number of recent ChIP-Seq experiments, performed in association with transcriptome analysis of gene knockout models have revealed that transcription factor binding is not always associated with changes in gene activity [34,35].

In contrast to the endogenous mouse *α-globin* promoter, we have shown that KLF3 does appear to regulate the expression of a human transgenic promoter in erythroid cells. The transgene is driven by a minimal human *α-globin* promoter and *HS-40* and perhaps this subset of elements is more reliant on repression by KLF3 than the entire set of globin regulatory elements. In the case of the endogenous *α-globin* locus, chromatin conformation capture experiments suggest that gene expression is dependent upon chromosomal looping of distal enhancers to the proximal promoter, in a process that is dependent upon many regulatory factors [17]. The removal of such complexity in the transgene most likely offers a far greater opportunity for observing the contribution that single factors make to expression levels. Alternatively, it should be noted that the experiments presented here primarily analyzed KLF3 function in murine cells, and thus it remains possible that KLF3 may play a role in *α-globin* regulation in human erythroid cells. Indeed, the related factor KLF4 has been shown to positively regulate the human *α-globin* promoter in reporter assays and to drive the endogenous *HBA* gene in K562 cells [36].

The up-regulation of GFP expression in Line3::*Klf3*^−/−^ mice shows that KLF3 can functionally repress the transgenic *α-globin* regulatory sequences *in vivo,* and may function as an epigenetic modifier of transgene expression. KLF3 mediates repression of its target genes by binding the co-repressor CtBP [4], which in turn recruits a repressive complex that includes several epigenetic modifiers, such as LSD1, G9A, EUHMT, PC2, HDAC1, and HDAC2 [37,38]. These factors facilitate histone methylation, demethylation and deacetylation, and are responsible for the addition of repressive epigenetic marks and gene silencing. It is possible that the absence of KLF3 in Line3::*Klf3*^−/−^ erythrocytes prevents CtBP from being recruited to the transgene, and it is this that allows the rewriting of epigenetic marks permissive for transcription, resulting in the up-regulation of GFP expression. Indeed, the Line3 mice have frequently been used in ENU mutagenesis screens for modulators of variegated expression, and these screens have predominantly culminated in the identification of epigenetic modifiers, including HDAC1 [39-43].

Another possible explanation for the lack of de-repression of the endogenous *α-globin* gene in red blood cells is that the locus is already fully open and maximally expressed, so significant further de-repression cannot occur. In contrast, the transgene contains only a limited subset of regulatory sequences, and may therefore be expressed at lower levels allowing its up-regulation in the absence of KLF3. To circumvent this, we examined regulation in murine embryonic fibroblasts, as *α-globin* mRNA expression is limited to low but detectable levels in this cell type. In these non-erythroid cells, we identified a modest but significant increase in *α-globin* gene expression in the absence of KLF3. Moreover, in support of a role for CtBP in the regulation of the *α-globin* locus, we note that another group have observed a similar de-repression (4-fold) of *α-globin* gene expression from microarray analysis of *Ctbp*^−/−^ murine embryonic fibroblasts [44].

Both the human and mouse *α-globin* loci lie in an open chromosomal region, surrounded by a number of actively expressed genes and in non-erythroid cells these loci retain the hallmarks of constitutively accessible chromatin [45]. This contrasts significantly with the more isolated *β-globin* gene cluster, where in non-erythroid cells a silent heterochromatic state is established and maintained. It therefore appears that the *α-globin* locus employs different silencing mechanisms to prevent expression in non-red blood cells. In the case of the human locus, this is achieved by targeted recruitment of the repressive polycomb complex, PRC2, to CpG islands in the promoter regions [45]. However, these CpG islands have been significantly eroded in the murine *α-globin* locus (Figure 2) and recruitment of PRC2 has not been detected, most likely due to loss of polycomb recruitment sites [46]. The mechanism of *α-globin* gene silencing in non-erythroid tissue in the mouse therefore remains unclear. Here we suggest that KLF3 participates in this silencing and may do so not through direct interaction with the *α-globin* proximal promoter but via distal regulatory regions such as *HS-26*. In erythroid cells, *HS-26* is an enhancer element that loops to the *α-globin* promoter and is required for appropriate regulation of expression [17]. In non-erythroid cells, such looping is disrupted and occurs at a much lesser frequency [47]. Whilst these observations allude to the functional importance of the *HS-26* element, it should be noted that loss of *HS-26* only modestly deregulates *α-globin* expression in erythroid cells and has not been reported to perturb non-erythroid silencing [48,49]. Thus it is likely that correct tissue-specific control of the locus is achieved by a complex interplay between multiple *cis*-acting regulatory regions and positively- and negatively-acting *trans* factors such as KLF3 and KLF1.

## Conclusions

Excessive α-globin expression can be detrimental to cells and thus it is important that mechanisms exist to limit its expression. Collectively, the findings presented here suggest that the broadly expressed transcriptional repressor KLF3 may have a role in silencing the *α-globin* locus in some but not all contexts, and in particular in non-erythroid tissues. These results complement the previous observation that the KLF3 co-repressor CtBP is also required for the appropriate control of *α-globin* expression in non-erythroid cells [44].

## Competing interests

The authors declare that they have no competing interests.

## Authors’ contributions

RCMP, APWF and MC designed the study and wrote the manuscript. DV, KGRQ, DRH and EW coordinated and oversaw experiments, and assisted in manuscript preparation. RCMP performed FACS analysis. APWF, KSM and GEM conducted EMSA experiments. DV, WFL, BW, JB and KSM performed ChIP studies. CMA generated MEF cell lines. APWF and WFL conducted qRT-PCR. All authors have read, contributed to, and approved the final manuscript.
